# Laser-Assisted Microdissection

**Published:** 2008

**Authors:** Denesa Oberbeck

**Keywords:** Alcohol-related research, alcohol and other drug effects and consequences, brain, brain cells, brain function, brain structure, laser-assisted microdissection (LMD), laser capture, laser microbeam, reverse-transcription polymerase chain reaction (RT-PCR), expression profiling

When analyzing alcohol’s effects on the brain, researchers often want to look at small clusters of cells that can be studied in isolation from the surrounding brain tissue rather than at the entire brain or larger brain areas. This implies that relatively small numbers of cells have to be retrieved from the brain and studied in culture or subjected to biochemical analyses. The challenge then becomes how to isolate small numbers of cells from a specific brain region without including unwanted cells. One approach to solving this problem is to use a technology known as laser-assisted microdissection (LMD). This article reviews some of the principles of LMD and its use in alcohol research.

The first report of LMD was published by Meier-Ruge and colleagues in 1976. After that, the field developed steadily until two distinct microdissection technologies—laser capture (Emmert-Brick et al. 1996) and laser microbeam (Schutze et al. 1998)—were introduced in the 1990s. Although the nomenclature often is used interchangeably, there is a subtle but significant difference between the two systems:

- Laser capture technology utilizes a low-powered infrared laser to activate an adhesive thermoplastic film on the cap of a microcentrifuge tube that has been placed over the cells of interest. Through this activation, the film directly “captures” the cells on the cap, and they can then be analyzed further (see figure 7).
- Laser beam, or laser pressure catapulting, technology typically uses an ultraviolet laser beam directed through the microscope objectives to cut around cells of interest that are mounted on a membrane-coated glass slide. The excised cells then are either ejected or dropped via gravity into a receptacle (commonly a microcentrifuge tube), in which they can be studied further.

Initially developed for cancer research—in which it is imperative to separately analyze tumor cells and nearby normal tissue—the LMD techniques now are widely used throughout biomedical research. Because of the highly complex and heterogeneous nature of the brain, the technology is particularly suited for neuroscience research. Although researchers can distinguish different cell types based on their structure, electrophysiological properties, activity (i.e., expression) of marker genes, or production of specific proteins, it can be difficult to isolate a given population of cells for further analysis. Methods using manual dissection of brain regions result in an “averaging” of various cell types, so that it is impossible to determine whether any effects are unique to one cell type. LMD enables investigators to accurately dissect individual groups of brain cells (i.e., nuclei) or even cell types for subsequent analysis. For example, LMD followed by other biochemical analyses^1^ has been used to identify genes that are particularly abundant in three subregions of the amygdala of mice. This approach identified several genes that are enriched in the amygdala (Zerlinger and Anderson 2003). Prior studies using manually dissected whole-amygdala tissue had not been able to identify these genes because their level of expression was too low and undetectable among the expression of all the other genes active in the whole tissue.

Most researchers combine LMD with expression profiling. This means that they compare either the expression of a single gene between different cell types or under different environmental conditions (e.g., in the presence and absence of alcohol) or that they compare the activity levels of all active genes within a cell under different conditions. The activity levels of individual genes can be analyzed using a technique called reverse-transcription polymerase chain reaction (RT-PCR), whereas the activity levels (i.e., messenger RNA [mRNA] levels) of all active genes can be studied using microarray technology. In addition to analyzing isolated RNA, scientists also can study the DNA or the entirety of all proteins produced (i.e., the proteome) of cells isolated by LMD (Bohm et al. 2005). For example, LMD has been used to measure the effects of acute and chronic cocaine administration on the gene expression profiles of neurons from a brain region called the ventral tegmental area, which plays a central role in the reinforcing properties of alcohol and other drugs (Backes and Hemby 2003). Similarly, LMD has been used in alcohol-related research to isolate specific cell populations. For example, Chen and colleagues (2006) and Sakar and colleagues (2007) used LMD to study the effects of alcohol exposure on certain neurons in a cell group called the arcuate nucleus,^2^ which is located in the brain’s hypothalamus. Other investigators are beginning to use LMD to isolate specific neurons from a group of nerve cells called the Edinger-Westphal nucleus, which have been shown to be uniquely sensitive to the effects of alcohol.

## Future Outlook

**Figure 8:**
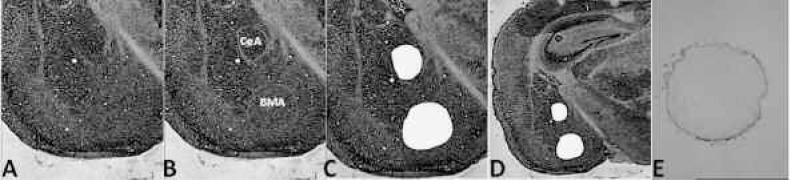
Representative photomicrographs showing a section of the mouse brain before and after laser microdissection of two subnuclei of the amygdala. **A)** Thionin-stained section of brain tissue viewed at 4x magnification before dissection. **B)** Computer graphic overlay (light grey circles) on photograph showing regions to be dissected. **C)** Section after microdissection; the nuclei have been removed. **D)** The same brain region at lower magnification (1.25x), showing the surrounding neuronal architecture. **E)** Microdissected region as seen isolated in a microcentrifuge tube. NOTE: BMA = basomedial nucleus of the amygdala; CeA = central nucleus of the amygdala.

As LMD becomes more widely used, laboratories should adopt some common quality-control procedures to ensure that indeed the desired cells have been isolated and no contamination with other cells occurs. For example, when trying to analyze cells from individual nuclei in the brain (e.g., subregions of the amygdala), researchers could measure the relative expression of three calcium-binding proteins called parvalbumin, calretinin, and calbindin. These proteins are produced in all brain regions, but their relative amounts differ among different regions or even nuclei. For example, if an experimental design requires dissecting (in the mouse brain) the central nucleus of the amygdala and the basomedial amygdala, the dissection of these two nuclei is relatively simple from an LMD perspective (see figure 8). However, subtle changes in dissection technique could lead to some substantial differences in the actual nature of the tissue acquired and presumably the results that are obtained. Therefore, as the experiment proceeds over time, with samples acquired from different animals, it will be critical to know that the dissection procedures have remained constant. At this point, biochemical expression analyses such as RT-PCR can be used to measure the expression of the calcium-binding proteins in each sample obtained. The expression of parvalbumin is relatively low in both the central and the basomedial nuclei of the amygdala; the expression of calretinin and calbindin, however, shows some variation between these nuclei. Moreover, the central nucleus can be further divided into three main divisions (lateral, medial, and capsular), which also show differential expression of the three genes. Thus, determining the relative expression of the three genes helps researchers determine exactly whether they have isolated the correct cells. With such quality-control strategies in place, LMD can help researchers gain valuable information about the functions and properties of very specific brain regions as well as about the effects that alcohol and other drugs have on these cells.

## Figures and Tables

**Figure 7:**
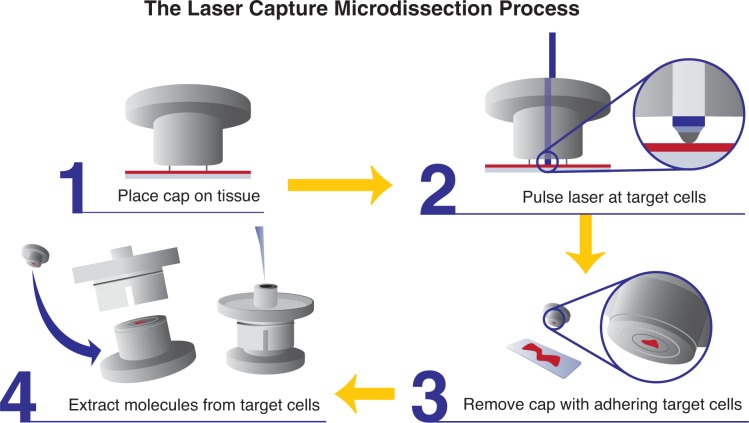
Schematic showing laser capture microdissection.

## Footnotes

1 For these analyses, RNA was isolated from the dissected cells, biochemically multiplied (i.e., amplified), and subsequently allowed to interact with and bind to (i.e., hybridize to) oligonucleotide microarrays.

2 These studies focused on those cells that produce a signaling molecule called pro-opiomelanocortin (POMC), which is further modified to the endogenous opioid, β endorphin. These neurons act on many brain areas and help mediate the effects of alcohol and other drugs as well as regulate appetite and sexual behavior.

## References

[b1-arh-31-3-249] Bachtell RK, Weitemier AZ, Galvan-Rosas A (2003). The Edinger-Westphal-lateral septum urocortin pathway and its relationship to alcohol consumption. Journal of Neuroscience.

[b2-arh-31-3-249] Backes E, Hemby SE (2003). Discrete cell gene profiling of ventral tegmental dopamine neurons after acute and chronic cocaine self-administration. Journal of Pharmacology and Experimental Therapeutics.

[b3-arh-31-3-249] Bohm C, Newrzella D, Sorgenfrei O (2005). Laser microdissection in CNS research. Drug Discovery Today.

[b4-arh-31-3-249] Dhaher R, Finn D, Snelling C, Hitzemann R (2008). Lesions of the extended amygdala in C57BL/6J mice do not block the intermittent ethanol vapor-induced increase in ethanol consumption. Alcoholism: Clinical and Experimental Research.

[b5-arh-31-3-249] Emmert-Buck MR, Bonner RF, Smith PD (1996). Laser capture microdissection. Science.

[b6-arh-31-3-249] Hitzemann B, Hitzemann R (1997). Genetic,s ethanol and the Fos response: A comparison of the C57BL/6J and DBA/2J inbred mouse strains. Alcoholism: Clinical and Experimental Research.

[b7-arh-31-3-249] Meier-Ruge W, Bielser W, Remy E (1976). The laser in the Lowry technique for microdissection of freeze-dried tissue slices. Histochemical Journal.

[b8-arh-31-3-249] Schutze K, Lahr G (1998). Identification of expressed genes by laser-mediated manipulation of single cells. Nature Biotechnology.

[b9-arh-31-3-249] Zirlinger M, Anderson D (2003). Molecular dissection of the amygdala and its relevance to autism. Genes, Brain, and Behavior.

